# Monitoring sustainable development goal 5.2: Cross-country cross-time invariance of measures for intimate partner violence

**DOI:** 10.1371/journal.pone.0267373

**Published:** 2022-06-17

**Authors:** Kathryn M. Yount, Irina Bergenfeld, Nishat Mhamud, Cari Jo Clark, Nadine J. Kaslow, Yuk Fai Cheong

**Affiliations:** 1 Hubert Department of Global Health and Department of Sociology, Emory University, Atlanta, GA, United States of America; 2 Hubert Department of Global Health, Rollins School of Public Health, Atlanta, GA, United States of America; 3 Department of Psychiatry and Behavioral Sciences, Emory University School of Medicine, Atlanta, GA, United States of America; 4 Department of Psychology, Emory University, Atlanta, GA, United States of America; Drexel University School of Public Health, UNITED STATES

## Abstract

**Background:**

The persistence and impacts of violence against women motivated Sustainable Development Goal (SDG) 5.2 to end such violence. Global psychometric assessment of cross-country, cross-time invariance of items measuring intimate partner violence (IPV) is needed to confirm their utility for comparing and monitoring national trends.

**Methods:**

Analyses of seven physical-IPV items included 377,500 ever-partnered women across 20 countries (44 Demographic and Health Surveys (DHS)). Analyses of five controlling-behaviors items included 371,846 women across 19 countries (42 DHS). We performed multiple-group confirmatory factor analysis (MGCFA) to assess within-country, cross-time invariance of each item set. Pooled analyses tested cross-country, cross-time invariance using DHSs that showed configural invariance in country-level multiple-group confirmatory factor analysis (MGCFAs). Alignment optimization tested approximate invariance of each item set in the pooled sample of all datasets, and in the subset of countries showing metric invariance over at least two repeated cross-sectional surveys in country-level MGCFAs.

**Results:**

In country-level MGCFAs, physical-IPV items and controlling-behaviors items functioned equivalently in repeated survey administrations in 12 and 11 countries, respectively. In MGCFA testing cross-country, cross-time invariance in pooled samples, neither item set was strictly equivalent; however, the physical-IPV items were approximately invariant. Controlling-behaviors items did not show approximate cross-country and cross-time invariance in the full sample or the sub-sample showing country-level metric invariance.

**Conclusion:**

Physical-IPV items approached approximate invariance across 20 countries and were approximately invariant in 11 countries with repeated cross-sectional surveys. Controlling-behaviors items were cross-time invariant within 11 countries but did not show cross-country, cross-time approximate invariance. Currently, the physical-IPV item set is more robust for monitoring progress toward SDG5.2.1, to end IPV against women.

## Introduction

One third of women experience intimate partner violence (IPV) in their lifetime [1]. IPV has a range of well-documented adverse effects on women’s mental health [2], physical health [3], and socioeconomic well-being [4], as well as effects on children [5] that perpetuate an inter-generational cycle of violence [6]. The global cost of IPV against women is more than $4.4 trillion or almost 5.2% of global gross domestic product [7].

The high prevalence, adverse effects, and persistence of IPV have motivated many calls to end violence against women particularly in their intimate relationships. A landmark commitment to end IPV against women was embodied in Sustainable Development Goal Target 5.2, which calls on national governments to “eliminate all forms of violence against all women and girls in public and private spheres, including trafficking and sexual and other types of exploitation” [8]. Indicator 5.2.1 is defined to measure the “proportion of ever-partnered women and girls aged 15 years and older subjected to physical, sexual or psychological violence by a current or former intimate partner in the previous 12 months, by form of violence and by age” [1].

The global commitment to monitor this indicator has generated a surge in research to understand the measurement properties of questionnaire modules assessing the major dimensions of IPV against women. Studies using survey data from 28 European Union (EU) countries have established the strict measurement invariance of measures for psychological [9], sexual [10], and physical [10] IPV. These studies relied on measures specific to the EU survey, which include more items for physical IPV (10 items), sexual IPV (4 items), controlling behaviors (8 items) and other psychological IPV (5 items) than used in other cross-national surveys that collect similar data. In low- and middle-income countries (LMICs), commonly used modules to measure IPV come from the World Health Organization (WHO) Multi-Country Study of Women’s Health and Domestic Violence [11] and the Demographic and Health Survey (DHS) Domestic Violence Module (DMV) [12]. The DHS DMV, which has aligned with the WHO IPV module, includes items to measure physical IPV (7 items) and controlling behaviors (5 items) in 89 DHS spanning 58 countries from 2005 to 2020. A recent analysis tested the cross-national invariance of the physical-IPV items and controlling-behaviors items for 36 countries for the period 2012–2018. Findings demonstrated approximate invariance of both item sets [13].

An important step to confirm the utility of these items for monitoring SDG 5.2.1 is to assess their *cross-*national and cross-time-invariance. Such an analysis would ascertain the measurement properties of these items both across countries and across repeated national surveys conducted with some periodicity. Evidence of the joint cross-national and cross-time invariance of these items would provide even stronger evidence for our capacity to monitor SDG 5.2.1. To date, the cross-national and cross-time invariance of these items is unknown, and the analysis presented here is designed to fill that critical gap. Our primary objective was to assess the cross-national and cross-time invariance of the seven DHS physical-IPV items, and separately, the five DHS controlling-behaviors items for the 20 and 19 countries, respectively, that had administered at least two repeated cross-sectional DHS approximately five years apart.

## Methods

### Sample and data on IPV

The DHS is a U.S. Agency for International Development (USAID)-funded program operating across more than 90 LMICs that collects data on population and health, including IPV among ever-partnered women. Per DHS protocols, between 15% and 100% of sampled households are administered a DVM, for which one woman 15–49 years in the household is randomly selected and interviewed (Table 1). To ensure similarity in the number and wording of items across administrations of the DHS, we restricted our sample to DVM versions V through VII, administered between 2005 and 2019. Within this frame, final samples included ever-partnered women 15–49 years from 19 countries and 18–49 years from one country in which at least two DHS were administered at intervals of 1 to 12 years with the same seven physical-IPV items (44 surveys total). For analyses of the controlling-behaviors items, two Rwanda surveys in the above sample were excluded; one survey did not administer the controlling behaviors items, bringing the number of countries to 19 and the number of surveys to 42.

**Table 1:** Countries, Demographic Health Survey samples, and intimate partner violence items included in time-invariance analysis.

Country	Survey year	Survey sampling and missingness										Survey training and design						
		Women’s questionnaire			Women’s Domestic Violence Module													
		Women eligible	Women inter-viewed	House-holds sampled	Women selected, inter-viewed	Women skipped PHY, CB items	Women eligible for PHY, CB items	Eligible women missing all PHY items		Eligible women missing all CB items		Enum-erator training, weeks	Field teams	Enum-erators	Report language	Trans-lated dialects	Prece-ding modules	Inter-view time, min
			n	%	N	n	n	n	%	n	%		n	n		#	#	
Cameroon	2011	All	15,426	50%	5,043	1,037	4,006	3	0.07%	3	0.07%	3	20	120	French	1	14	53.7
	2018	All	13,527	50%	6,682	1,992	4,690	0	0.00%	1	0.02%	4	17	136	French	1	10	56.3
Dominican Rep.	2007	All	27,195	50%	10,140	1,702	8,438	15	0.18%	17	0.20%	5	21	168	Spanish	NA	11	50.1
	2013	All	9,372	100%	6,996	1,193	5,803	3	0.05%	2	0.03%	4	12	108	Spanish	NA	10	44.4
Haiti	2005–06	All	10,575	50%	3,568	888	2,680	4	0.15%	3	0.11%	4	10	70	French	1	11	60.1
	2012	All	14,287	2/3	9,367	2,717	6,650	8	0.12%	8	0.12%	6	5	25	French	1	10	46.4
	2016–17	All	14,371	2/3	6,321	1,999	4,322	0	0.00%	0	0.00%	4	15	120	French	1	14	53.2
India	2005–06	All	124,385	N/F	83,703	14,219	69,484	48	0.07%	71	0.10%	2	NA	NA	English	18	9	58.9
	2015–16	All	699,686	15%	79,729	13,716	66,013	0	0.00%	46	0.07%	3	789	5523	English	17	13	55.3
Jordan	2012	EM	11,352	1/3	7,027	0	7,027	0	0.00%	0	0.00%	4	26	182	English	1	11	43.3
	2017–18	EM	14,689	2/3	6,852	0	6,852	0	0.00%	1	0.01%	5	27	162	English	1	12	41.3
Mali	2013	All	10,424	50%	3,459	339	3,120	0	0.00%	0	0.00%	5	25	125	French	3	13	57.3
	2018	All	10,519	50%	3,784	428	3,356	0	0.00%	2	0.06%	4	22	110	French	3	14	56.1
Malawi	2010	All	23,020	1/3	6,229	849	5,380	6	0.11%	5	0.09%	4	37	296	English	2	11	60.9
	2016–17	All	24,562	1/3	6,379	973	5,406	0	0.00%	0	0.00%	3	38	304	English	2	12	53.4
Mozambique	2011	All	13,754	1/3	6,835	1,011	5,824	0	0.00%	0	0.00%	6	26	182	Portuguese	NA	11	44.0
	2015	All^a^	7,749	1/3	3,690	333	3,357	483	14.39%	2	0.06%	6	25	NA	Portuguese	NA	9	41.5
Nigeria	2008	All	33,385	50%	23,752	4,363	19,389	147	0.76%	163	0.84%	3	37	296	English	3	12	66.4
	2013	All	34,948	50%	27,634	5,329	22,305	25	0.11%	120	0.54%	4	37	296	English	3	12	59.1
	2018	All	41,821	50%	10,678	1,768	8,910	0	0.00%	0	0.00%	4	37	296	English	3	12	56.4
Nepal	2011	All	12,674	50%	4,197	692	3,505	0	0.00%	0	0.00%	4	16	80	English	3	10	62.0
	2016	All	12,862	50%	4,444	618	3,826	0	0.00%	0	0.00%	3	16	80	English	3	12	76.6
Philippines	2008	All	13,594	100%	9,316	0^b^	9,316	838	9.00%	838	9.00%	2	57	456	English	6	10	68.4
	2013	All	16,155	100%	10,963	2,803	8,160	4	0.05%	3	0.04%	2	70	420	English	6	10	53.7
	2017	All	25,074	100%	17,968	4,753	13,215	0	0.00%	2	0.02%	5	90	360	English	6	11	45.0
Pakistan	2012–13	EM	13,558	1/3	3,687	0	3,687	1	0.03%	22	0.60%	3	20	120	English	2	9	51.8
	2017–18	EM	12,634	1/3	4,085	0	4,085	0	0.00%	10	0.24%	4	22	132	English	2	11	56.9
Rwanda	2010^c^	All	13,671	50%	5,008	1,532	3,476	6	0.17%	3,476	100.00%	3	15	105	English	2	11	62.3
	2014–15	All	13,497	50%	2,679	771	1,908	1	0.05%	1	0.05%	4	17	119	English	1	11	68.0
Sierra Leone	2013	All	16,658	50%	5,185	870	4,315	6	0.14%	13	0.30%	4	24	144	English	2	12	60.4
	2019	All	15,574	50%	5,248	1,193	4,055	0	0.00%	0	0.00%	2	24	168	English	4	14	55.1
Senegal	2018	All	9,414	50%	1,957	451	1,506	0	0.00%	0	0.00%	1	5	30	French	6	14	39.0
	2019	All	8,649	50%	1,865	397	1,468	0	0.00%	0	0.00%	1	5	30	French	6	14	36.9
Tajikistan	2012	All	9,656	100%	5,547	1,142	4,405	3	0.07%	43	0.98%	3	14	84	English	2	10	55.8
	2017	All	10,718	100%	6,353	1,040	5,313	0	0.00%	6	0.11%	4	14	84	English	2	12	31.9
Timor-Leste	2009–10	All	13,137	1/3	2,951	789	2,162	0	0.00%	0	0.00%	4	13	78	English	2	11	60.6
	2016	All	12,607	2/3	5,122	1,428	3,694	0	0.00%	0	0.00%	4	20	120	English	1	15	47.1
Uganda	2006	All	8,531	1/3	2,087	338	1,749	1	0.06%	2	0.11%	4	15	105	English	6	10	75.0
	2011	All	8,674	1/3	2,056	351	1,705	3	0.18%	2	0.12%	4	16	112	English	7	10	63.8
	2016	All	18,506	1/3	9,232	1,696	7,536	0	0.00%	0	0.00%	4	21	147	English	8	12	62.6
Zambia	2013–14	All	14,773	100%	11,778	2,362	9,416	2	0.02%	3	0.03%	5	24	240	English	7	11	61.6
	2018	All	13,683	100%	9,503	2,145	7,358	0	0.00%	0	0.00%	3	22	154	English	7	13	59.7
Zimbabwe	2010–11	All	9,171	100%	6,542	1,260	5,282	2	0.04%	6	0.11%	2	15	120	English	2	11	50.5
	2015	All	9,955	100%	7,223	1,423	5,800	0	0.00%	0	0.00%	2	15	120	English	2	12	59.7

Abbreviations: PHY, physical items; CB, controlling behaviors items; NA, not applicable; EM, ever married.

^a^Mozambique sampled women 18–59 years old instead of 15–49 years old in 2015 survey.

^b^In Philippines, all women were eligible to interview for physical and controlling behaviors items in the 2008 survey. "Never married women/has a boyfriend or dating partner" not skipped.

^c^Rwanda did not ask controlling behaviors items in 2010 survey.

The total sample included 380,012 women who were selected and administered the DVM and were not skipped out of the IPV items due to never-partnered status. Of these, 2,512 were missing data on all physical-IPV items, bringing the final analytic sample to 377,500 ever-partnered women across 20 countries and 44 DHS in analyses of the physical IPV items. The final analytic sample for controlling- behaviors items was 374,628 women across 19 countries and 42 DHS. Removal of individuals with missing and “don’t know” responses for all controlling behaviors items brought the analytical sample for controlling behaviors to 371,846. All DHS samples were downloaded with written permission from the DHS program.

For items on physical, sexual, and psychological IPV, participants across all DHS were asked whether their husband or partner had ever done each act (see S1 Table for item wordings). Participants who responded yes were asked whether their husband or partner had done the act often, sometimes, or never within the past 12 months. For controlling behaviors, participants were asked whether their husband or partner did or did not do each of the behaviors without reference to a time frame. We elected to use the lifetime rather than the past-12-month timeframe for responses to the physical-IPV items for greater comparability across the two item sets. In a subset of countries, the DHS also included a maximum of three sexual-IPV items and a maximum of three psychological-IPV items (S1 Table). We did not use these item sets in our final analyses due to their questionable content validity relative to uniform definitions of these constructs [14, 15] and the small number of included items [16].

### Analytic strategy

In step 1 we tested the measurement invariance of the set of seven physical-IPV items, and separately, the five controlling-behaviors items, over repeated cross-sectional surveys within each country. We performed multiple group confirmatory factor analysis (MGCFA) using weighted least squares estimation, comparing the fit of configural models, in which all loadings and thresholds were estimated freely across repeated cross-sectional surveys, and scalar models, in which all loadings and thresholds were constrained to be equal across repeated cross-sectional surveys. For countries with three repeated cross-sectional surveys, we performed invariance testing across each combination of two surveys. We used DHS-generated probability weights and cluster variables in all models to account for selection probabilities and clustering. We used several indices to assess the fit of configural models: chi-square (χ^2^), Root Mean Square Error of Approximation (RMSEA, adequate fit ≤0.08, good fit ≤0.05), and Comparative Fit Index and Tucker-Lewis Index (CFI, TLI, ≥0.95) [17]. We used the χ^2^ difference test to assess invariance over repeated cross-sectional surveys [18, 19].

In step 2, we conducted a pooled analysis of all DHSs that showed configural invariance in each individual-country MGCFA. In step 3, we used MGCFA with maximum likelihood (ML) estimation to assess metric invariance across repeated cross-sectional surveys within each country and in a pooled analysis across all DHSs. When pooled analyses showed a lack of evidence for metric invariance, we used the alignment optimization (AO) approach in step 4 to perform an approximate invariance test in the pooled sample of all datasets. This approach relaxes some assumptions of MGCFA by allowing estimated country-specific model parameters to vary from the estimated model parameters in the pooled dataset following a normal distribution. The criterion for approximate invariance is evidence that ≤25% of model parameters (loadings and thresholds) are non-invariant. In step 5, where approximate invariance was not supported in the pooled sample of all datasets, we restricted the sample to countries that showed metric invariance over repeated cross-sectional surveys in individual-country analyses. We then reran alignment optimization using this subset of surveys and countries. We used STATA 17 [20] for data cleaning and management. All measurement invariance testing was performed in MPlus 8 [21].

## Results

### Characteristics of included surveys

Survey characteristics, including logistics and design, are summarized across the full sample of 44 surveys (Table 1). The duration of enumerator training varied across surveys from between one to six weeks, with most surveys (43%) conducting training in four weeks. Across all surveys, data collection was conducted by an average of 42 field teams. The total number of survey field teams ranged from five in Senegal to 789 in India. Most surveys (91%) were translated into at least one local dialect. India had the most translations, into 17–18 dialects. All surveys included three sensitive modules on HIV, contraception, and sexual activity that preceded the DVM. The DVM typically was the last module in the women’s questionnaire, with at least nine modules preceding it. Nearly half of the surveys (21 of 44) reported a mean duration of the women’s interview of 45 to 60 minutes; however, the interview duration ranged from 31.9 minutes to 76.6 minutes.

Surveys were administered between 2005 and 2019; 16% were administered in 2018. Within countries, the average number of years between repeated survey administrations was five. To create the DVM sample, all surveys selected between 15% and 100% of households interviewed in the main DHS, and then sampled one woman per household for the DVM. A plurality of surveys (39%) sampled 50% of interviewed households to create the household sample for the DVM. On average, 10,520 women across surveys were selected and interviewed for the DVM; however, sample sizes for the DVM ranged from 1,865 women in the 2019 Senegal DHS to 83,703 women in the 2005–06 India DHS. In most surveys (91%), both ever-married and never-married women were eligible for the DVM. Two surveys in Jordan and two surveys in Pakistan interviewed only ever-married women for the DVM. Among the surveys that interviewed all women for the DVM, only ever-married women and women who ever lived with a man were eligible for the physical IPV and the controlling behaviors items. However, in the 2008 Philippines DHS, women who have (had) a boyfriend or dating partner previously or at the time of the survey were eligible for the physical-IPV and controlling-behaviors items. All surveys interviewed women ages 15 to 49 years for the DVM, except the 2015 Mozambique DHS, which included women 18 to 59 years.

### Country-specific and pooled time invariance of physical IPV items and controlling behaviors items

Of the 20 countries included in the measurement-invariance testing of the seven physical-IPV items, all showed good fit for the individual-country configural model across at least two DHS administrations (Table 2). According to changes-in-fit-statistics criteria (ΔCFI, ΔTLI), individual-country models showed scalar invariance over time; however, according to the χ2 difference test between scalar and configural models, individual-country models for only five countries showed scalar invariance over time. In metric invariance testing using maximum likelihood estimation, 12 countries had a non-significant likelihood ratio test across repeated DHS administrations in individual-country analyses, suggesting metric invariance (Table 3).

**Table 2:** Scalar invariance testing for Demographic Health Survey physical intimate partner violence items (n = 20 countries) and controlling behaviors Items (n = 19 countries).

Country	Survey year	Range of loadings	Model	RMSEA	95% CI LL	95% CI UL	χ2	df	P-value	CFI	TLI	delta RMSEA	delta CFI	delta χ2
**Physical IPV items**														
Cameroon	2011	0.863–1.057	Configural	0.029	0.024	0.034	127.807	28	<0.0001	0.995	0.992			
	2018	0.993–1.055	Scalar	0.029	0.024	0.034	153.636	33	<0.0001	0.994	0.992			
	Scalar	0.872–1.067	Configural vs Scalar				30.112	5	<0.0001			<0.001	0.001	25.829
Dominican Rep.	2007	0.927–1.106	Configural	0.015	0.011	0.020	75.827	28	<0.0001	0.999	0.998			
	2013	0.911–1.021	Scalar	0.016	0.012	0.020	90.420	33	<0.0001	0.999	0.998			
	Scalar	0.924–1.009	Configural vs Scalar				17.112	5	0.0043			0.001	<0.001	14.593
Haiti	2005–06	0.893–1.000	Configural	0.013	0.008	0.018	73.916	42	0.0017	0.999	0.999			
	2012	0.816–1.018	Scalar	0.012	0.008	0.017	88.900	52	0.0011	0.999	0.999			
	2016–17	0.756–1.041	Configural vs Scalar				17.640	10	0.0613			0.001	<0.001	14.984
	Scalar	0.873–1.000												
India	2005–06	0.845–1.021	Configural	0.022	0.021	0.024	980.229	28	<0.0001	0.997	0.996			
	2015–16	0.880–1.044	Scalar	0.020	0.019	0.021	949.445	33	<0.0001	0.997	0.996			
	Scalar	0.839–1.017	Configural vs Scalar				40.294	5	<0.0001			0.002	<0.001	-30.784
Jordan	2012	0.989–1.012	Configural	0.021	0.018	0.026	117.628	28	<0.0001	0.997	0.996			
	2017–18	0.847–1.032	Scalar	0.020	0.016	0.023	121.531	33	<0.0001	0.997	0.996			
	Scalar	0.989–1.019	Configural vs Scalar				7.635	5	0.1775			0.001	<0.001	3.903
Mali	2013	0.881–1.092	Configural	0.016	0.009	0.023	51.231	28	0.0047	0.995	0.992			
	2018	0.779–1.119	Scalar	0.015	0.008	0.021	56.835	33	0.0061	0.994	0.993			
	Scalar	0.884–1.155	Configural vs Scalar				8.204	5	0.1453			0.001	0.001	5.604
Malawi	2010	0.935–1.065	Configural	0.015	0.010	0.020	63.862	28	0.001	0.999	0.998			
	2016–17	0.954–1.057	Scalar	0.015	0.010	0.019	71.680	33	0.001	0.999	0.999			
	Scalar	0.948–1.065	Configural vs Scalar				10.093	5	0.0726			<0.001	<0.001	7.818
Mozambique	2011	0.954–1.117	Configural	0.017	0.012	0.023	65.170	28	0.0001	0.997	0.995			
	2015	0.916–1.053	Scalar	0.018	0.013	0.023	79.529	33	<0.0001	0.996	0.995	0.001	0.001	
	Scalar	0.911–1.090	Configural vs Scalar				16.523	5	0.0055					14.359
Nigeria	2008	0.928–1.000	Configural	0.020	0.018	0.022	336.693	42	<0.0001	0.997	0.995			
	2013	0.829–10.44	Scalar	0.020	0.018	0.022	408.669	52	<0.0001	0.996	0.995			
	2018	0.870–1.030	Configural vs Scalar				85.407	10	<0.0001			<0.001	0.001	71.976
	Scalar	0.905–1.000												
Nepal	2011	0.829–1.010	Configural	0.005	0.000	0.014	30.129	28	0.3571	1.000	1.000			
	2016	0.913–1.070	Scalar	0.011	0.001	0.018	47.711	33	0.047	1.000	1.000			
	Scalar	0.831–1.019	Configural vs Scalar				16.598	5	0.0053			0.006	<0.001	17.582
Philippines	2008	0.946–1.049	Configural	0.013	0.010	0.016	115.664	42	<0.0001	0.999	0.998			
	2013	0.933–1.049	Scalar	0.012	0.009	0.015	127.819	52	<0.0001	0.999	0.999			
	2017	0.941–1.054	Configural vs Scalar				19.291	10	0.0367			0.001	<0.001	12.155
	Scalar	0.945–1.045												
Pakistan	2012–13	0.767–1.029	Configural	0.017	0.011	0.023	60.474	28	0.0004	0.999	0.998			
	2017–18	0.943–1.007	Scalar	0.017	0.012	0.023	70.912	33	0.0001	0.999	0.998			
	Scalar	0.788–1.029	Configural vs Scalar				13.199	5	0.0216			<0.001	<0.001	10.438
Rwanda	2010	0.927–1.081	Configural	0.054	0.048	0.060	248.828	28	<0.0001	0.992	0.988			
	2014–15	0.897–1.014	Scalar	0.093	0.088	0.099	806.870	33	<0.0001	0.971	0.963			
	Scalar	0.905–1.078	Configural vs Scalar				492.568	5	<0.0001			0.039	0.021	558.042
Sierra Leone	2013	0.859–1.087	Configural	0.035	0.030	0.040	173.142	28	<0.0001	0.991	0.986			
	2019	0.861–1.041	Scalar	0.032	0.027	0.037	174.866	33	<0.0001	0.991	0.989			
	Scalar	0.884–1.101	Configural vs Scalar				15.531	5	0.0083			0.003	<0.001	1.724
Senegal	2018	0.981–1.130	Configural	0.053	0.045	0.062	147.169	28	<0.0001	0.961	0.942			
	2019	0.726–1.080	Scalar	0.049	0.042	0.058	153.019	33	<0.0001	0.961	0.950			
	Scalar	0.922–1.085	Configural vs Scalar				11.876	5	0.0365			0.004	<0.001	5.85
Tajikistan	2012	0.986–1.018	Configural	0.030	0.026	0.035	152.925	28	<0.0001	0.993	0.990			
	2017	0.834–1.116	Scalar	0.028	0.024	0.033	160.838	33	<0.0001	0.993	0.991			
	Scalar	0.945–1.024	Configural vs Scalar				22.227	5	0.0005			0.002	<0.001	7.913
Timor-Leste	2009–10	0.965–1.131	Configural	0.037	0.031	0.043	139.980	28	<0.0001	0.986	0.979			
	2016	0.953–1.224	Scalar	0.045	0.039	0.050	226.314	33	<0.0001	0.976	0.970			
	Scalar	1.000–1.148	Configural vs Scalar				77.954	5	<0.0001			0.008	0.010	86.334
Uganda	2006	0.917–1.034	Configural	0.030	0.025	0.034	176.836	42	<0.0001	0.997	0.995			
	2011	0.861–1.034	Scalar	0.028	0.024	0.032	201.916	52	<0.0001	0.996	0.995			
	2016	0.886–1.111	Configural vs Scalar				30.827	10	0.0006			0.002	0.001	25.08
	Scalar	0.899–1.051												
Zambia	2013–14	0.965–1.063	Configural	0.022	0.018	0.025	137.386	28	<0.0001	0.997	0.995			
	2018	0.939–1.070	Scalar	0.022	0.019	0.025	165.593	33	<0.0001	0.996	0.995			
	Scalar	0.968–1.081	Configural vs Scalar				32.673	5	<0.0001			<0.001	0.001	28.207
Zimbabwe	2010–11	0.878–1.044	Configural	0.021	0.016	0.025	94.213	28	<0.0001	0.995	0.996			
	2015	0.879–1.077	Scalar	0.016	0.012	0.021	81.205	33	<0.0001	0.997	0.997			
	Scalar	0.878–1.044	Configural vs Scalar				1.649	5	0.8952			0.005	0.002	-13.008
Pooled	Configural	0.755–1.204	Configural	0.022	0.021	0.023	3210.190	616	<0.0001	0.997	0.996			
	Scalar	1.000–1.104	Scalar	0.032	0.032	0.033	8281.467	831	<0.0001	0.992	0.991			
			Configural vs Scalar				4888.360	215	<0.0001			0.01	0.005	5071.277
**Controlling behaviors items**														
Cameroon	2011	0.799–1.000	Configural	0.073	0.065	0.081	242.246	10	<0.0001	0.968	0.937			
	2018	0.892–1.000	Scalar	0.065	0.058	0.072	252.242	13	<0.0001	0.967	0.950			
	Scalar	0.808–1.000	Configural vs Scalar				7.139	3	0.0676			0.008	0.001	9.996
Dominican Rep.	2007	0.985–1.295	Configural	0.044	0.038	0.051	150.256	10	<0.0001	0.990	0.981			
	2013	0.687–1.000	Scalar	0.050	0.044	0.055	239.973	13	<0.0001	0.985	0.976			
	Scalar	0.982–1.313	Configural vs Scalar				91.686	3	<0.0001			0.006	0.005	89.717
Haiti	2005–06	0.830–1.097	Configural	0.072	0.066	0.079	373.494	15	<0.0001	0.982	0.965			
	2012	0.844–1.000	Scalar	0.063	0.058	0.069	402.209	21	<0.0001	0.981	0.973			
	2016–17	0.893–1.035	Configural vs Scalar				23.145	6	0.0007			0.009	0.001	28.715
	Scalar	0.825–1.076												
India	2005–06	0.690–1.151	Configural	0.046	0.044	0.048	1454.066	10	<0.0001	0.964	0.928			
	2015–16	0.978–1.171	Scalar	0.049	0.047	0.051	2110.484	13	<0.0001	0.948	0.920			
	Scalar	0.725–1.058	Configural vs Scalar				672.934	3	<0.0001			0.003	0.016	656.418
Jordan	2012	1.000–2.008	Configural	0.047	0.041	0.053	162.997	10	<0.0001	0.966	0.932			
	2017–18	1.000–1.531	Scalar	0.040	0.035	0.046	158.682	13	<0.0001	0.968	0.950			
	Scalar	1.000–1.877	Configural vs Scalar				6.842	3	0.0771			0.007	0.002	-4.315
Mali	2013	0.958–1.085	Configural	0.077	0.068	0.087	203.03	10	<0.0001	0.973	0.945			
	2018	0.939–1.025	Scalar	0.071	0.063	0.080	227.948	13	<0.0001	0.970	0.953			
	Scalar	0.974–1.074	Configural vs Scalar				23.683	3	<0.0001			0.006	0.003	24.918
Malawi	2010	0.892–1.040	Configural	0.056	0.049	0.063	178.907	10	<0.0001	0.982	0.965			
	2016–17	0.892–1.053	Scalar	0.049	0.043	0.056	183.566	13	<0.0001	0.982	0.973			
	Scalar	0.894–1.032	Configural vs Scalar				3.902	3	0.2723			0.007	<0.001	4.659
Mozambique	2011	1.000–1.376	Configural	0.066	0.058	0.074	200.372	10	<0.0001	0.975	0.950			
	2015	0.942–1.016	Scalar	0.061	0.054	0.068	223.542	13	<0.0001	0.972	0.958			
	Scalar	1.000–1.377	Configural vs Scalar				25.435	3	<0.0001			0.005	0.003	23.17
Nigeria	2008	1.000–1.235	Configural	0.056	0.053	0.060	811.367	15	<0.0001	0.972	0.945			
	2013	1.000–1.260	Scalar	0.052	0.050	0.055	988.564	21	<0.0001	0.966	0.952			
	2018	1.000–1.111	Configural vs Scalar				172.525	6	<0.0001			0.004	0.006	177.197
	Scalar	1.000–1.245												
Nepal	2011	0.833–1.110	Configural	0.034	0.025	0.043	51.587	10	<0.0001	0.990	0.981			
	2016	0.830–1.027	Scalar	0.033	0.025	0.041	65.019	13	<0.0001	0.988	0.981			
	Scalar	0.800–1.089	Configural vs Scalar				13.839	3	0.0031			0.001	0.002	13.432
Philippines	2008	0.918–1.050	Configural	0.035	0.031	0.040	200.875	15	<0.0001	0.993	0.987			
	2013	0.980–1.068	Scalar	0.032	0.028	0.035	228.493	21	<0.0001	0.992	0.989			
	2017	0.897–1.038	Configural vs Scalar				42.104	6	<0.0001			0.003	0.001	27.618
	Scalar	0.921–1.040												
Pakistan	2012–13	1.000–1.221	Configural	0.051	0.043	0.060	110.105	10	<0.0001	0.986	0.972			
	2017–18	1.000–1.143	Scalar	0.045	0.038	0.053	116.639	13	<0.0001	0.985	0.978			
	Scalar	1.000–1.246	Configural vs Scalar				6.464	3	0.0911			0.006	0.001	6.534
Sierra Leone	2013	0.744–1.000	Configural	0.085	0.077	0.093	308.786	10	<0.0001	0.963	0.926			
	2019	0.743–1.000	Scalar	0.085	0.078	0.093	409.053	13	<0.0001	0.951	0.924			
	Scalar	0.726–1.000	Configural vs Scalar				111.702	3	<0.0001			<0.001	0.012	100.267
Senegal	2018	0.921–1.023	Configural	0.028	0.012	0.045	21.934	10	0.0154	0.998	0.996			
	2019	1.000–1.192	Scalar	0.027	0.012	0.041	26.626	13	0.014	0.998	0.996			
	Scalar	0.906–1.029	Configural vs Scalar				5.389	3	0.1454			0.001	<0.001	4.692
Tajikistan	2012	1.000–1.258	Configural	0.051	0.044	0.059	137.738	10	<0.0001	0.982	0.963			
	2017	0.851–1.101	Scalar	0.048	0.041	0.055	156.663	13	<0.0001	0.979	0.968			
	Scalar	1.000–1.215	Configural vs Scalar				19.825	3	0.0002			0.003	0.003	18.925
Timor-Leste	2009–10	0.836–1.030	Configural	0.044	0.034	0.054	66.233	10	<0.0001	0.984	0.968			
	2016	0.887–1.086	Scalar	0.039	0.030	0.048	70.044	13	<0.0001	0.984	0.975			
	Scalar	0.816–1.036	Configural vs Scalar				7.047	3	0.0704			0.005	<0.001	3.811
Uganda	2006	0.886–1.000	Configural	0.087	0.080	0.094	429.241	15	<0.0001	0.968	0.936			
	2011	0.918–1.037	Scalar	0.072	0.066	0.078	424.206	21	<0.0001	0.969	0.956			
	2016	0.935–1.010	Configural vs Scalar				18.845	6	0.0044			0.015	0.001	-5.035
	Scalar	0.904–1.000												
Zambia	2013–14	0.807–1.000	Configural	0.067	0.061	0.073	387.008	10	<0.0001	0.978	0.956			
	2018	0.820–1.000	Scalar	0.061	0.056	0.066	420.481	13	<0.0001	0.976	0.963			
	Scalar	0.806–1.000	Configural vs Scalar				23.167	3	<0.0001			0.006	0.002	33.473
Zimbabwe	2010–11	1.000–1.218	Configural	0.094	0.087	0.101	496.196	10	<0.0001	0.962	0.923			
	2015	1.000–1.155	Scalar	0.088	0.082	0.094	567.668	13	<0.0001	0.956	0.933			
	Scalar	1.000–1.208	Configural vs Scalar				66.838	3	<0.0001			0.006	0.006	71.472
Pooled	Configural	0.688–1.773	Configural	0.055	0.054	0.057	5931.688	210	<0.0001	0.977	0.954			
	Scalar	1.000–1.275	Scalar	0.063	0.062	0.063	11883.202	333	<0.0001	0.953	0.941			
			Configural vs Scalar				6229.611	123	<0.0001			0.008	0.024	5951.514

Abbreviations: RMSEA, root mean square error of approximation; CI LL, confidence interval lower limit; CL UL, confidence interval upper limit; χ2, chi-square; df, degrees of freedom; CFI, comparative fit index; TLI, Tucker-Lewis index; IPV, intimate partner violence.

**Table 3:** Metric invariance testing for Demographic Health Survey physical intimate partner violence items (n = 20 countries) and controlling behaviors items (n = 19 countries).

Country	Model	n	Surveys	LL	#FP	SC	AIC	BIC	CAIC	SABIC	LR Test	P-value
**Physical IPV items**												
Cameroon	Configural	8693	2	-21753.427	29	2.2332	43564.853	43769.891	43650.08992	43677.734	6.701351799	0.082051099
	Metric	8693	2	-21765.556	26	2.0732	43583.113	43766.94	43659.53041	43684.316		
Dominican Rep.	Configural	14223	2	-24864.067	29	2.9484	49786.134	50005.45	49877.57075	49913.29	3.85619371	0.277417172
	Metric	14223	2	-24872.773	26	2.7676	49797.546	49994.174	49879.52377	49911.548		
Haiti	Configural	13640	3	-28170.219	44	2.5272	56428.438	56759.352	56566.36983	56619.524	5.361989043	0.718277744
	Metric	13640	3	-28179.762	36	2.2978	56431.525	56702.272	56544.37732	56587.867		
India	Configural	135449	2	-295913.063	29	3.9788	591884.126	592168.8	592003.9475	592076.637	1.982899836	0.575963483
	Metric	135449	2	-296012.713	26	3.8186	592077.426	592332.651	592184.8522	592250.022		
Jordan	Configural	13879	2	-24579.184	29	3.2982	49216.368	49434.973	49307.49639	49342.814	5.963218826	0.113413486
	Metric	13879	2	-24598.088	26	2.9472	49248.176	49444.167	49329.87731	49361.542		
Mali	Configural	6476	2	-14037.878	29	2.1444	28133.757	28330.257	28215.2839	28238.102	2.129188965	0.546030245
	Metric	6476	2	-14041.472	26	2.0023	28134.944	28311.116	28208.03798	28228.495		
Malawi	Configural	10780	2	-21437.191	29	2.0762	42932.383	43143.661	43020.32794	43051.502	2.832652248	0.418152944
	Metric	10780	2	-21442.875	26	1.8527	42937.749	43127.171	43016.59809	43044.546		
Mozambique	Configural	8698	2	-17129.776	29	1.9731	34317.552	34522.606	34402.79516	34430.449	46.93127527	3.59459E-10
	Metric	8698	2	-17187.241	26	1.9182	34426.483	34610.325	34502.9069	34527.702		
Nigeria	Configural	50432	3	-97794.073	44	2.1413	195676.146	196064.595	195839.0651	195924.762	309.4740552	3.95951E-62
	Metric	50432	3	-98189.194	36	2.0497	196450.388	196768.209	196583.6854	196653.801		
Nigeria 6 & 7	Configural	31190	2	-46213.045	29	1.9082	92484.09	92726.178	92585.41645	92634.016	11.43960513	0.00957147
	Metric	31190	2	-46227.955	26	1.8276	92507.909	92724.953	92598.7544	92642.326		
Nepal	Configural	7331	2	-14114.409	29	3.4175	28286.818	28486.914	28369.90773	28394.758	55.02453379	6.78392E-12
	Metric	7331	2	-14505.218	26	2.1728	29062.435	29241.832	29136.93024	29159.209		
Philippines	Configural	29849	3	-55195.564	44	1.9738	110479.127	110844.499	110632.0249	110704.668	12.62342774	0.125479894
	Metric	29849	3	-55210.317	36	1.893	110492.633	110791.574	110617.7315	110677.167		
Pakistan	Configural	7771	2	-15252.546	29	2.7708	30563.092	30764.879	30646.91583	30672.723	54.15437013	1.04014E-11
	Metric	7771	2	-15472.503	26	2.1532	30997.005	31177.917	31072.1584	31095.294		
Rwanda	Configural	5377	2	-14939.923	29	1.3562	29937.846	30128.953	30017.03166	30036.8	11.33255994	0.010057038
	Metric	5377	2	-14954.576	26	1.2143	29961.152	30132.489	30032.14604	30049.87		
Sierra Leone	Configural	8364	2	-23203.258	29	2.5583	46464.517	46668.436	46549.26601	46576.279	10.13833359	0.017426119
	Metric	8364	2	-23223.84	26	2.385	46499.68	46682.504	46575.66276	46599.881		
Senegal	Configural	2974	2	-4265.149	29	1.9561	8588.298	8762.23	8660.024888	8670.086	1.939232373	0.585114548
	Metric	2974	2	-4267.986	26	1.8442	8587.973	8743.912	8652.278865	8661.3		
Tajikistan	Configural	9715	2	-18033.507	29	2.1031	36125.015	36333.276	36211.64984	36241.119	13.56424513	0.003562493
	Metric	9715	2	-18055.829	26	1.966	36163.659	36350.376	36241.33151	36267.752		
Timor-Leste	Configural	5856	2	-13266.74	29	1.8658	26591.48	26785.062	26671.74043	26692.908	13.66153255	0.003404012
	Metric	5856	2	-13288.057	26	1.721	26628.115	26801.671	26700.07163	26719.05		
Uganda	Configural	10986	3	-32862.605	44	1.859	65813.211	66134.603	65947.00694	65994.777	11.42016683	0.179009195
	Metric	10986	3	-32876.193	36	1.7433	65824.387	66087.344	65933.85623	65972.941		
Zambia	Configural	16772	2	-41442.736	29	2.5253	82943.473	83167.569	83036.98496	83075.409	2.293822362	0.513705411
	Metric	16772	2	-41447.831	26	2.3041	82947.661	83148.576	83031.50121	83065.949		
Zimbabwe	Configural	11080	2	-23572.433	29	1.9324	47202.867	47414.941	47291.15765	47322.782	3.056222707	0.383037711
	Metric	11080	2	-23578.032	26	1.7326	47208.065	47398.2	47287.22203	47315.575		
Pooled	Configural	378345	44	-1698186.888	659	2.8563	3397691.776	3404837.683	3400708.604	3402743.349	1779.274599	6.1097E-245
	Metric	378345	44	-1700481.882	446	2.9884	3401855.764	3406691.993	3403897.502	3405274.583		
Pooled*	Configural	274801	27	-1091479	404	2.9031	2183765.446	2188017.063	2185559.363	2186733.132	450.7598296	1.57229E-37
	Metric	274801	27	-1092038.924	276	3.0973	2184629.849	2187534.418	2185855.017	2186657.277		
**Controlling behaviors items**												
Cameroon	Configural	8691	2	-27534.788	21	2.5002	55111.576	55260.046	55173.29646	55193.312	0.476756975	0.489894957
	Metric	8691	2	-27536.912	20	2.1797	55113.823	55255.224	55172.60539	55191.667		
Dominican Rep.	Configural	14220	2	-41501.959	21	3.5182	83045.918	83204.728	83112.12889	83137.992	101.5174018	7.08384E-24
	Metric	14220	2	-41842.357	20	3.3588	83724.713	83875.961	83787.77199	83812.403		
Haiti	Configural	13638	3	-45843.611	32	2.6139	91751.221	91991.881	91851.53402	91890.188	11.67033439	0.019978746
	Metric	13638	3	-45865.354	28	2.455	91786.707	91997.285	91874.48102	91908.303		
Haiti 6 & 7	Configural	8474	2	-24559.171	21	2.9197	49160.342	49308.282	49221.83186	49241.548	43.78329094	3.66823E-11
	Metric	8474	2	-24696.031	20	2.7531	49432.062	49572.957	49490.62377	49509.4		
India	Configural	135355	2	-351233.483	21	5.634	702508.966	702715.095	702595.727	702648.356	47.08554234	6.79551E-12
	Metric	135355	2	-351562.093	20	5.2178	703164.185	703360.499	703246.8155	703296.938		
Jordan	Configural	13878	2	-37117.03	21	3.6836	74276.059	74434.358	74342.04886	74367.622	3.717191793	0.053854875
	Metric	13878	2	-37142.466	20	3.1835	74324.932	74475.693	74387.77854	74412.135		
Mali	Configural	6474	2	-19955.353	21	2.4189	39952.705	40094.992	40011.74063	40028.259	1.530781541	0.215995187
	Metric	6474	2	-19960.566	20	2.1993	39961.132	40096.643	40017.35545	40033.088		
Malawi	Configural	10779	2	-31257.278	21	2.1937	62556.556	62709.549	62620.24015	62642.813	0.655558207	0.418132457
	Metric	10779	2	-31259.613	20	1.9472	62559.226	62704.933	62619.87757	62641.376		
Mozambique	Configural	8693	2	-24692.202	21	2.4582	49426.405	49574.881	49488.12656	49508.146	11.84954583	0.000576754
	Metric	8693	2	-24736.165	20	2.2101	49512.33	49653.735	49571.11339	49590.179		
Nigeria	Configural	50225	3	-153988.059	32	3.8346	308040.117	308322.494	308158.5474	308220.797	37.63011849	1.33573E-07
	Metric	50225	3	-154075.867	28	3.7157	308207.733	308454.813	308311.3598	308365.828		
Nigeria 6 & 7	Configural	31091	2	-81476.32	21	3.7868	162994.64	163169.878	163067.9853	163103.14	8.714105026	0.003157574
	Metric	31091	2	-81513.524	20	3.5492	163067.047	163233.941	163136.9007	163170.381		
Nepal	Configural	7331	2	-16120.561	21	3.2189	32283.121	32428.018	32343.29043	32361.285	3.592830144	0.058029338
	Metric	7331	2	-16134.201	20	3.0002	32308.403	32446.4	32365.70526	32382.844		
Philippines	Configural	29847	3	-78469.996	32	2.3503	157003.992	157269.715	157115.1888	157168.019	7.22736211	0.124350011
	Metric	29847	3	-78481.206	28	2.2429	157018.412	157250.919	157115.7092	157161.936		
Pakistan	Configural	7740	2	-16097.607	21	2.8248	32237.214	32383.251	32297.87756	32316.518	0.47093973	0.492555166
	Metric	7740	2	-16099.798	20	2.5008	32239.597	32378.68	32297.37082	32315.124		
Sierra Leone	Configural	8355	2	-27107.616	21	3.2625	54257.232	54404.874	54318.59288	54338.14	9.863754128	0.001685668
	Metric	8355	2	-27156.158	20	2.9335	54352.315	54492.928	54410.75493	54429.371		
Senegal	Configural	2974	2	-5520.619	21	2.6063	11083.238	11209.189	11135.17816	11142.464	1.743245111	0.186728174
	Metric	2974	2	-5528.816	20	2.2664	11097.632	11217.585	11147.09882	11154.037		
Tajikistan	Configural	9669	2	-28096.694	21	2.768	56235.388	56386.098	56298.08101	56319.364	2.018278751	0.155415226
	Metric	9669	2	-28104.644	20	2.5125	56249.287	56392.821	56308.99563	56329.264		
Timor-Leste	Configural	5853	2	-15252.926	21	2.3856	30547.852	30688.021	30605.96695	30621.289	5.552017513	0.018459398
	Metric	5853	2	-15275.244	20	2.1029	30590.489	30723.983	30645.83557	30660.429		
Uganda	Configural	10983	3	-38186.143	32	2.1872	76436.285	76670.017	76533.58907	76568.325	5.253146564	0.262295098
	Metric	10983	3	-38196.306	28	1.9469	76448.612	76653.127	76533.75219	76564.147		
Zambia	Configural	16770	2	-52234.963	21	2.9629	104511.927	104674.201	104579.6412	4607.464	7.51937079	0.0061039
	Metric	16770	2	-52263.931	20	2.7258	104567.862	104722.409	104632.3527	104658.85		
Zimbabwe	Configural	11076	2	-33020.809	21	2.1021	66083.618	66237.181	66147.55004	66170.446	1.373747854	0.241168993
	Metric	11076	2	-33026.37	20	1.8024	66092.739	66238.99	66153.62766	66175.432		
Pooled	Configural	372551	42	-1912269.375	461	3.4859	3825460.75	3830452.518	3827568.067	3828987.438	2220.691018	0
	Metric	372551	42	-1915454.157	340	3.7057	3831588.313	3835269.877	3833142.517	3834189.341		
Pooled*	Configural	119442	24	-600213.109	263	2.915	1200952.219	1203500.843	1202024.51	1202665.019	381.6870626	1.38824E-45
	Metric	119442	24	-600745.244	196	2.9583	1201882.489	1203781.844	1202681.611	1203158.949		

Abbreviations: LL, likelihood; #FP, number of free parameters; SC, scaling correction factor; AIC, Akaike information criterion; BIC, Bayesian information criterion; CAIC, consistent Akaike information criterion; SABIC, sample-size adjusted BIC; LR Test, likelihood ratio test; IPV, intimate partner violence.

*Pooled countries showing metric invariance in individual country models.

In a pooled analysis of all 20 countries, while configural invariance was evident, neither metric (Table 3) nor scalar (Table 2) invariance was achieved according to difference testing. Changes in fit statistics, however, did not provide evidence of non-invariance in the scalar models. When the pooled sample was restricted to the 27 DHSs (from 12 countries) that showed evidence of metric invariance across repeated DHS administrations in within-country analyses, metric invariance still was not evident (Table 3).

In analyses of the five controlling-behaviors items, all 19 individual-country analyses showed good fit of the configural model (Table 2). Six countries showed evidence of scalar invariance over time according to χ2 difference testing, with 11 showing evidence of metric invariance according to the likelihood ratio test in maximum likelihood models (Table 3). For the pooled sample of 19 countries, neither metric nor scalar invariance was suggested by the likelihood ratio test or the χ2 difference test, respectively. Metric invariance was not evident in the 11-country pooled sample for which repeated cross-sectional DHSs showed metric invariance over time in individual-country analyses. Neither the individual-country nor pooled analyses showed evidence of non-invariance according to changes in fit statistics in weighted least squares models.

### Tests of approximate invariance of physical-IPV items and controlling-behaviors items

Table 4 presents the AO-based results, in which we assessed approximate measurement invariance separately for the seven physical-IPV items (Panel 1) and the five controlling-behaviors items (Panel 2). For physical IPV, 118 (or 38% of) estimated thresholds, 44 (or 14% of) estimated loadings, and 26% of all parameter estimates were measurement non-invariant (S2 Table). For controlling behaviors, 132 (or 61% of) estimated thresholds, 78 (or 36% of) estimated loadings, and 49% of all parameter estimates were measurement non-invariant. A guideline of 25% or fewer total non-invariant parameter estimates is recommended for trustworthy latent mean estimates and their comparison across groups. The results suggested that neither item set exhibited approximate measurement invariance across the 20 countries and repeated DHS administrations. Among the seven physical-IPV items, the item ‘slap’ had a low degree of threshold and loading invariance, as shown by its low R^2^ (Table 4).

**Table 4:** Thresholds, loadings, and R^2^ values from alignment optimization analysis of physical intimate partner violence items and controlling behaviors items using the full pooled sample of Demographic and Health Surveys.

**Panel A. Results from alignment optimization analysis for physical Items, n = 378,345 across Demographic Health Surveys in 20 countries, 2006–2019**				
**Items**	**Thresholds**		**Loadings**	
	**Weighted average value across invariant groups**	**R** ^**2**^	**Weighted average value across invariant groups**	**R** ^**2**^
Push you, shake you, or throw something at you?	2.25	0.589	2.946	0.346
Slap you?	0.09	0.011	3.511	0.00
Punch with his fist or with something that could hurt you?	3.362	0.528	3.217	0.618
Kick you, drag you, or beat you up?	4.473	0.662	3.11	0.635
Try to choke you or burn you on purpose?	5.916	0.546	2.599	0.509
Threaten to attack you with a knife, gun or other weapon?	5.85	0.459	2.602	0.511
Twist your arm or pull your hair?	3.585	0.756	2.919	0.546
# (%) of threshold non-invariant parameters = 118 (38)				
# (%) of loading non-invariant parameters = 44 (14)				
# (%) of total non-invariant parameters = 162 (26)				
**Panel B. Results from alignment optimization analysis for controlling behaviors Items, n = 372, 692 across Demographic Health Surveys in 19 countries, 2006–2019**				
**Items**	**Thresholds**		**Loadings**	
	**Weighted average value across invariant groups**	**R** ^**2**^	**Weighted average value across invariant groups**	**R** ^**2**^
Is jealous or angry if she talks to other men?	-1.653	0.759	2.457	0.179
Frequently accuses her of being unfaithful?	1.116	0.846	2.359	0.309
Does not permit her to meet her female friends?	1.266	0.706	2.835	0.406
Tries to limit her contact with her family?	3.422	0.627	2.984	0.426
Insists on knowing where she is at all times?	-0.216	0.824	1.921	0.594
# (%) of threshold non-invariant parameters = 132 (61)				
# (%) of loading non-invariant parameters = 78 (36)				
# (%) of total non-invariant parameters = 210 (49)				

Table 5 presents the AO-based results in which we assessed approximate measurement invariance separately for the physical-IPV items and the controlling-behaviors items for a subset of countries that displayed metric invariance across at least two administrations of the DHS (Table 5). For physical IPV, 61 (or 36% of) estimated thresholds, 15 (or 9% of) estimated loadings, and 20% of all parameter estimates were measurement non-invariant. For controlling behaviors, 47 (or 39% of) estimated thresholds, 21 (or 17.5% of) estimated loadings, and 28% of all parameter estimates were measurement non-invariant. Thus, the results suggested that DHS physical-IPV items but not the controlling-behaviors items exhibited approximate measurement invariance across countries and repeated administrations showing within-country metric invariance and allowed acceptable alignment performance. Additionally, the R^2^ values showed that all seven physical-IPV items had a reasonable degree of threshold and loading invariance (Table 5).

**Table 5:** Thresholds, loadings, and R^2^ values from alignment optimization analysis of physical IPV items and controlling behaviors items using the subsetted pooled sample of Demographic and Health Surveys.

**Panel A. Results from alignment optimization analysis for physical IPV items, n = 274,801 across Demographic Health Surveys in 12 countries, 2006–2019**				
**Items**	**Thresholds**		**Loadings**	
	**Weighted average value across invariant groups**	**R** ^**2**^	**Weighted average value across invariant groups**	**R** ^**2**^
Push you, shake you, or throw something at you?	1.525	0.698	3.24	0.553
Slap you?	0.164	0.799	3.267	0.625
Punch with his fist or with something that could hurt you?	2.813	0.656	3.301	0.732
Kick you, drag you, or beat you up?	4.011	0.831	3.389	0.547
Try to choke you or burn you on purpose?	4.566	0.631	2.574	0.712
Threaten to attack you with a knife, gun or other weapon?	5.757	0.355	2.093	0.52
Twist your arm or pull your hair?	3.005	0.795	2.962	0.604
# (%) of threshold non-invariant parameters = 61 (36)				
# (%) of loading non-invariant parameters = 15 (9)				
# (%) of total non-invariant parameters = 76 (20)				
**Panel B. Results from alignment optimization analysis for controlling behaviors Items, n = 119,442 across Demographic Health Surveys in 11 countries, 2006–2019**				
**Items**	**Thresholds**		**Loadings**	
	**Weighted average value across invariant groups**	**R** ^**2**^	**Weighted average value across invariant groups**	**R** ^**2**^
Is jealous or angry if she talks to other men?	-1.073	0.789	1.654	0.482
Frequently accuses her of being unfaithful?	1.183	0.866	1.782	0.586
Does not permit her to meet her female friends?	1.567	0.233	2.209	0.113
Tries to limit her contact with her family?	2.785	0.756	2.208	0.52
Insists on knowing where she is at all times?	0.059	0.881	1.374	0.515
# (%) of threshold non-invariant parameters = 47 (39)				
# (%) of loading non-invariant parameters = 21 (17.5)				
# (%) of total non-invariant parameters = 68 (28)				

## Discussion

### Summary of findings

Testing of within-country cross-time measurement invariance, relevant for national efforts to monitor IPV trends using the DHS DVM, revealed that the seven physical-IPV items and the five controlling-behaviors items functioned equivalently in repeated survey administrations within a subset of LMIC countries. In the second stage, we examined cross-country and cross-time invariance in pooled samples including multiple countries with two or more survey administrations each. While these two item sets were not strictly equivalent in these samples, the physical-IPV item set exhibited approximate invariance over time and across countries in a restricted sample of countries exhibiting within-country, cross-time metric invariance of the item set. The five controlling-behaviors items did not meet the recommended threshold for non-invariant parameters to infer approximate invariance across time and across countries. A prior analysis found evidence of approximate invariance for physical-IPV and controlling-behaviors item sets across 36 DHS administered in 36 countries during 2012–2018 [13, 22]. The present analysis corroborates that the physical-IPV items function comparably across time and across very diverse national contexts and highlights the cross-time invariance of the controlling-behaviors items within selected countries. Evidence of greater threshold than loading non-invariance, especially for controlling-behaviors items, suggests greater comparability in item interpretation across contexts and time, but less comparability in the likelihood of endorsing items (responding yes to acts of IPV) across contexts and time.

### Limitations and strengths

Findings should be interpreted considering the study’s limitations and strengths. The study assessed measurement properties of item sets in the DHS; therefore, findings cannot be extended to item sets that measure other forms of IPV nor to item sets that are used in other, non-DHS IPV survey modules. However, the DHS are widely administered across LMICs and represent approximately half the data being reported to monitor progress toward SDG5.2.1. It is the single largest contributor to SDG5.2.1 monitoring and has IPV items like those used in WHO surveys, making it possibly the most important source for rigorous psychometric testing. Findings reported here may represent a best-case scenario. The DHS program provides technical support for survey administration, which, while not entirely uniform across countries or time periods (Table 1), does provide a level of consistency in administration that does not exist across the wide variety of survey formats and forms of administration that represent the data pool available for SDG5.2.1 monitoring. This level of consistency bolsters its use for research, but potentially limits study findings to the item sets tested using similarly consistent methods of administration. Finally, in pooled analyses, we were unable to account for possible auto-correlation across national surveys within countries. Despite their limitations, the findings are based on 44 DHS conducted in 20 diverse countries spanning four regions (Africa, Asia, Latin America, and the Middle East) and 15 years (2005–2019).

### Implications for research and policy

These findings suggest the seven DHS physical-IPV items are promising for comparing and monitoring national trends in IPV toward achieving SDG5.2.1, to eliminate IPV against women. The low R^2^ for the item ‘slap’ in AO analysis suggests the potential benefit of focused cognitive testing of this item across diverse contexts to improve its measurement properties, and thereby, the item set as a whole. The five DHS controlling-behaviors items, in their present formulation, show promise in some countries for monitoring within-country trends in this form of IPV; however, their lack of approximate invariance in full and restricted pooled samples of countries with repeated DHS administrations caution against their use to compare and to monitor national trends in this form of IPV toward achieving SDG5.2.1. Cognitive testing of these items, and psychometric testing of a revised controlling-behaviors item set in diverse, multi-country samples of women, may improve their measurement properties and their utility for monitoring SDG5.2.1 cross-nationally and over time.

Improved global measures of controlling behaviors also will improve our estimates of the impacts of these forms of IPV on the health of victims and their children worldwide, providing insights into strategies for prevention and response. These advances are critical, given that controlling behaviors in an intimate partnership often indicate more severe forms of IPV. Improved measurement of controlling behaviors is motivated further by changes in some criminal codes to include ‘controlling or coercive behaviors’ as prosecutable offenses [23]. Thus, promoting standard, contextually informed, definitions of controlling behaviors and enhancing the measurement properties of controlling-behaviors items will strengthen the capacity for cross-national monitoring of trends, and may stimulate changes to other national criminal codes to include controlling behaviors as a prosecutable offense. Such changes would provide new legal norms about the nature and scope of IPV and new mechanisms to deter controlling behaviors [24]. Such changes also offer the potential to move away from a narrow focus on physical injury towards emotional and psychological trauma in criminal cases of IPV [23], expanding response options for victims of these forms of IPV.

This analysis did not assess the cross-country and cross-time measurement properties of DHS item sets measuring psychological IPV (typically 3 items) and sexual IPV (typically 2–3 items). Presently, these DHS item sets align only narrowly with uniform definitions of these forms of IPV [15, 25], suggesting a notable lack of content validity. Still, there may be practical benefit in future analyses to assess the psychometric properties of these limited item sets to establish an evidence-base regarding the extent of their cross-country and cross-time measurement invariance. The current lack of content validity of these item sets, however, has important practical implications for interpreting trends in these forms of violence. Namely, the current content of the item sets implies that only certain underlying ranges of these forms of IPV are observable. As a result, estimated trends in these forms of IPV—even if they are shown to be measurement invariant—may inaccurately capture true underlying trends. For example, if reductions in sexual IPV using measured physical tactics occurs alongside increases in sexual IPV using unmeasured non-physical tactics, observed rates of sexual IPV will appear to decline, when, trends in the totality of sexual IPV are stable or increasing. Indeed, focused studies using more comprehensive measures confirm the high levels of forms of sexual IPV [26] and psychological IPV [27] that the DHS does not measure. Hence, expanding these item sets is needed to capture the full range of relevant behaviors for accurate monitoring of SDG5.2.1. Such an effort need not result in large item sets, because the process of psychometric assessment can identify a precise subset that is reasonably content valid. Therefore, we recommend desk reviews of validated instruments and qualitative research in diverse settings to generate expanded item pools for sexual and psychological IPV, cognitive testing of these expanded item pools, repeated cross-cultural pilot surveys, and rigorous psychometric assessment to identify item sets that are content valid and measurement invariant across-context and across-time. Such an effort would round out the much-needed evidence to identify a common, validated item pool for inclusion in national surveys of violence against women. Agencies like the United Nations (UN), national governments, and global donors would have the evidence needed to make maximally informed decisions about the allocation of resources to prevent and to respond to IPV, based on trends in all domains of IPV that are optimally measured.

## Conclusion

This analysis is the most comprehensive assessment of the global cross-country cross-time invariance of seven physical-IPV items and five controlling-behaviors items. While measures of controlling behaviors, psychological IPV, and sexual IPV are improved, the physical IPV items are reasonable for monitoring trends in IPV against women to guide resources for effective prevention and response.

## Supporting information

S1 Table Item sets capturing physical, sexual, and psychological intimate partner violence from the Domestic Violence Module for the Demographic Health Survey versions 5 to 7. (PDF)

S2 Table Invariant thresholds and loadings from alignment optimization analysis of physical intimate partner violence items and controlling behaviors items using the full and subsetted pooled samples of Demographic Health Surveys. (PDF)
