# Supplementary material for: Monitoring sustainable development goal 5.2: Cross-country cross-time invariance of measures for intimate partner violence
Source: PLoS One. 2022 Jun 17;17(6):e0267373. doi: 10.1371/journal.pone.0267373 (PMC9205513; doi:10.1371/journal.pone.0267373)
Supplement: S1 Table — (PDF) [file pone.0267373.s001.pdf]

S1 Table. Item sets capturing physical, sexual, and psychological intimate partner violence from the Domestic Violence Module for the Demographic Health Survey Versions 5 to 7.

**Physical intimate partner violence: Did your husband or partner ever…**
(1) Push you, shake you, or throw something at you?
(2) Slap you?
(3) Twist your arm or pull your hair?
(4) Punch you with his fist or with something that could hurt you?
(5) Kick you, drag you, or beat you up?
(6) Try to choke you or burn you on purpose?
(7) Threaten or attack you with a knife, gun, or other weapon?
**Emotional intimate partner violence: Did your husband or partner ever…**
(1) Say or do something to humiliate you in front of others?
(2) Threaten to hurt or harm you or someone you care about?
(3) Insult you or make you feel bad about yourself?
**Sexual intimate partner violence: Did your husband or partner ever…**
(1) Physically force you to have sexual intercourse with him when you did not want to?
(2) Force you to perform any other sexual acts you did not want to?
(3) Force you with threats or in any other way to perform sexual acts you did not want to?
**Controlling Behaviours:**
(1) He is/was jealous or angry if you talk/talked to other men?
(2) He frequently accuses/accused you of being unfaithful?
(3) He does/did not permit you to meet your female friends?
(4) He tries/tried to limit your contact with your family?
(5) He insists/insisted on knowing where you are/were at all times?
