# Supplementary material for: Monitoring sustainable development goal 5.2: Cross-country cross-time invariance of measures for intimate partner violence
Source: PLoS One. 2022 Jun 17;17(6):e0267373. doi: 10.1371/journal.pone.0267373 (PMC9205513; doi:10.1371/journal.pone.0267373)
Supplement: S2 Table — (PDF) [file pone.0267373.s002.pdf]

S2 Table. Invariant thresholds and loadings from alignment optimisation analysis of physical intimate partner violence items and controlling behaviours items using the full and subsetted pooled samples of Demographic Health Surveys.

		Full sample				Subsample with metric invariance			
		Physical items (n=44 surveys)		Controlling behaviours (n=42 surveys)		Physical items (n=27 surveys)		Controlling behaviours (n=24 surveys)	
**Country**	**Year**	**Invariant thresholds**	**Invariant loadings**	**Invariant thresholds**	**Invariant loadings**	**Invariant thresholds**	**Invariant loadings**	**Invariant thresholds**	**Invariant loadings**
Cameroon	2011	3	1	4	0	1	1	0	1
	2018	2	0	2	3	2	2	1	2
Dominican Rep.	2007	3	1	4	4	2	0	NA	NA
	2013	5	0	4	2	2	0	NA	NA
Haiti	2005-06	3	0	2	2	2	0	NA	NA
	2012	3	0	2	2	2	0	NA	NA
	2016-17	3	1	2	1	2	0	NA	NA
India	2005-06	4	1	5	4	5	1	NA	NA
	2015-16	4	4	4	4	5	2	NA	NA
Jordan	2012	3	1	2	2	0	0	3	1
	2017-18	3	1	2	2	2	0	2	2
Mali	2013	2	1	3	1	1	0	4	0
	2018	2	0	3	0	3	0	1	0
Malawi	2010	3	2	4	0	3	1	2	0
	2016-17	4	0	4	1	3	0	2	1
Mozambique	2011	1	1	4	3	NA	NA	NA	NA
	2015	1	1	3	1	NA	NA	NA	NA
Nigeria	2008	5	2	2	2	NA	NA	NA	NA
	2013	3	2	5	2	NA	NA	NA	NA
	2018	2	2	4	4	NA	NA	NA	NA
Nepal	2011	1	2	3	2	NA	NA	2	1
	2016	1	1	4	2	NA	NA	2	1
Philippines	2008	3	1	3	1	2	1	3	1
	2013	3	2	4	2	2	2	2	2
	2017	3	2	4	3	2	1	1	2
Pakistan	2012-13	2	1	3	1	NA	NA	2	1
	2017-18	0	0	3	2	NA	NA	3	0
Rwanda	2010	4	1	NA	NA	NA	NA	NA	NA
	2014-15	2	0	NA	NA	NA	NA	NA	NA
Sierra Leone	2013	2	1	2	2	NA	NA	NA	NA
	2019	3	0	2	2	NA	NA	NA	NA
Senegal	2018	2	1	2	0	1	0	2	0
	2019	2	1	1	1	1	0	1	0
Tajikistan	2012	2	0	4	1	NA	NA	3	0
	2017	2	3	3	2	NA	NA	3	0
Timor-Leste	2009-10	1	0	2	0	NA	NA	NA	NA
	2016	2	1	4	1	NA	NA	NA	NA
Uganda	2006	4	0	4	1	1	0	1	1
	2011	4	0	2	2	2	0	1	0
	2016	4	1	4	5	3	1	3	2
Zambia	2013-14	3	2	1	3	2	1	NA	NA
	2018	3	1	4	2	4	1	NA	NA
Zimbabwe	2010-11	3	1	4	2	3	0	1	2
	2015	3	1	4	1	3	1	2	1
Total		118	44	132	78	61	15	47	21

NA, not applicable.
